# Impact of interventions on work-related outcomes for individuals with musculoskeletal injuries after road traffic crash: a systematic review protocol

**DOI:** 10.1186/s13643-019-1178-2

**Published:** 2019-10-29

**Authors:** Charlotte L. Brakenridge, Elise M. Gane, Esther J. Smits, Nicole E. Andrews, Venerina Johnston

**Affiliations:** 1 RECOVER Injury Research Centre, The University of Queensland, Brisbane, QLD Australia; 2 Occupational Therapy Department and The Professor Tess Cramond Multidisciplinary Pain Centre, The Royal Brisbane and Women’s Hospital, Brisbane, QLD Australia

**Keywords:** Musculoskeletal injury, Whiplash, Pain, Road traffic crash, Traffic accidents, Return to work, Work, Employment, Intervention, Systematic review

## Abstract

**Background:**

Musculoskeletal injuries are the most common non-fatal injury from road traffic crashes. Even when the injuries are mild, they can cause pain which can affect return to work rates and work ability post-crash. Workplace output losses are the biggest cost from traffic crash-related injuries. There is a need to identify effective interventions that can improve work-related outcomes (e.g. time to return to work, sick leave, and work ability) in this group and a need to understand the intervention components, external factors, and participant characteristics that may be associated with improvement.

**Methods:**

A systematic review will be conducted using seven databases and search terms related to road traffic crash, musculoskeletal injury, work-related outcomes, and study design. Intervention studies will be eligible if they report on at least one work-related outcome, include adults with a traffic crash-related musculoskeletal injury (e.g. fracture or whiplash), include a comparison group, and are written in English. Interventions can be medical, therapeutic, work-based, multicomponent, or other. Two researchers will independently screen titles and abstracts, review full texts for inclusion in the review, and perform the data extraction. The main outcomes of the review will be time until return to work and duration of sick leave. The results will be narratively described, with meta-analyses conducted where possible.

**Discussion:**

This review will explore the effectiveness of interventions in individuals with traffic crash-related musculoskeletal injury on work-related outcomes and will act as a useful source for researchers, policy makers, and stakeholders when developing and implementing interventions in this group.

**Systematic review registration:**

PROSPERO CRD42018103746

## Background

As many as 50 million people are injured in road traffic crashes worldwide each year [1]. In Australia, there were approximately 32,000 hospitalised and 224,000 non-hospitalised injuries from road traffic crashes in 2016 [2]. Musculoskeletal injuries (e.g. fractures [1] and whiplash [3]) are the most common non-fatal injury from road traffic crash; other injuries include traumatic brain injury, open wounds, internal injuries, and spinal cord injuries [1].

Injury costs from road traffic crashes in Australia (i.e. not including fatalities and property damage) were calculated to be over $13 billion in 2016 [2]. The main cost from traffic crash-related injury is in workplace output losses [4], including loss in contributions to the economy, replacing staff who do not return to work, and staff returning to work at reduced hours [4]. Musculoskeletal injuries from a road traffic crash, even those classed as ‘mild’ [5], can result in persistent pain [6–8] which may impact on staying and returning to work and working optimally. This is evidenced in studies reporting extended periods of sick leave [9] and return to work rates as low as 18% after 26 weeks [10] in people with whiplash-associated disorder. Work ability is also impaired in people with whiplash-associated disorder compared with the general population [11].

The health benefits of work are well recognised [12], and returning to work is a rehabilitation priority after a road traffic crash [13, 14] likely to assist with overall recovery [15]. While there have been systematic reviews evaluating determinants and interventions for returning to work after traumatic brain injury [16–19] and spinal cord injury [20–22], less is known about the effectiveness of interventions to improve return to work rates and other work-related outcomes (e.g. sick leave, work ability) in individuals with musculoskeletal injuries after road traffic crash. Previous systematic reviews (conducted prior to 2011 and which focused on general recovery) have suggested that early interdisciplinary treatment (e.g. psychosocial support and physiotherapy within 6 months post-injury) may result in a higher rate of return to work [23] and that postural exercises delivered by a physiotherapist (compared to standard intervention) may reduce sick leave after whiplash [24]. However, these conclusions were only based on two individual studies [25, 26], and the primary focus of these reviews was not on work-related outcomes and only addressed one specific musculoskeletal problem [23, 24]. Thus, given the large proportion of survivors of road traffic crash with a range of musculoskeletal injuries, and the potential detrimental impact of these injuries on returning to work and working optimally, a targeted systematic review of the intervention literature is needed to evaluate the impact of interventions on work-related outcomes in individuals with traffic crash-related musculoskeletal injury.

It is also important to evaluate the content of the interventions and the context in which the interventions are delivered. Intervention factors that may plausibly have an effect on results include intervention length, who delivered the intervention, and the interventionists’ frequency of contact with participants. Using an ecological model such as Bronfenbrenner’s Systems Theory [27], the key contextual factors to consider include the microsystem (e.g. the individual person), the mesosystem (e.g. the workplace), and the macrosystem (e.g. government legislation). At the individual level, self-rated health [28], disability level [29], recovery expectations [28, 29], job type [28, 30], and work hours [28] of injured individuals have been associated with return to work rates after a road traffic crash and may also be associated with return to work rates and other work-related outcomes following an intervention.

At the workplace level, better work-related outcomes may be associated with targeting the workplace directly. Work-based interventions (e.g. job redesign, adaptation of working hours) have been effective at reducing time to return to work and sick leave in workers with musculoskeletal disorders compared to usual care [31]. In addition, evidence across countries and compensation schemes indicates that work-based interventions may be more effective at improving work-related outcomes than medical/therapeutic interventions (e.g. surgery, medication, or exercise therapy) in workers with low back pain [32]. In individuals injured in a road traffic crash, social support from employers has also been beneficially associated with returning to work [33, 34]. Thus, interventions that include employers or target ways to address low levels of workplace support may be associated with improved return to work outcomes after road traffic crash. At the macro level, differences in compensation regulation and legislation across and within countries can impact on returning to work [32, 35]. Specifically, less rigid compensation schemes are associated with better return to work rates [32, 35].

Concurrent changes in other outcomes after intervention, such as pain and cognition, are also important to consider. Interventions that aim to reduce pain or improve cognition may improve work-related outcomes through achieving better functioning at home and/or at work. In one prospective study, less self-reported bodily pain 6 weeks after whiplash injury was associated with improved return to work outcomes after 12 months [36]. Concentration issues and cognitive dysfunction have also been associated with greater work disability after road traffic crash in two prospective cohort studies [37, 38]. Identifying concurrent changes in other outcomes will assist in the understanding of changes in work-related outcomes.

Thus, the objectives of this systematic review are (1) to evaluate the impact of interventions (medical, therapeutic, work-based, multicomponent, or other) on work-related outcomes in individuals who have sustained a traffic crash-related musculoskeletal injury; (2) to understand the intervention components, participant characteristics (sociodemographic, health-, injury- and work-related), workplace characteristics, and external factors (e.g. compensation schemes) that may be associated with improvement in work-related outcomes; and (3) to evaluate the impact of these interventions on secondary outcomes (e.g. pain).

## Methods

This systematic review protocol follows the Preferred Reporting Items for Systematic review and Meta-Analysis Protocols (PRISMA-P) statement [39] (see Additional file 1) and has been registered with the PROSPERO database (CRD42018103746).

### Inclusion criteria

Studies will be included in the review if they meet the following criteria.

#### Participants

Participants in the studies will be adults (≥ 18 years) who have sustained a musculoskeletal injury (e.g. whiplash, fractures, dislocations, sprains) after a road traffic crash. Participants cannot be children and cannot have a neurological injury of the spine or brain (i.e. spinal cord injury or traumatic brain injury). Participants can have non-catastrophic neurological issues if it is secondary to their primary musculoskeletal injury (e.g. peripheral nerve injuries). Studies including both children and adults will only be included if the results are reported separately for adults. Studies including a range of injuries from road traffic crashes will only be included if the results are reported separately for participants with musculoskeletal injuries. Studies of the same injury but of different causes (e.g. road traffic crash and other accidents) will be included as long as the road traffic crash injuries are the majority (i.e. at least ~ 80%) of injury cases. Paper authors will be contacted (twice via email) if there are a combination of injuries in the sample and these have not been reported separately, or if it is unclear from the paper whether the injuries occurred from a road traffic crash.

#### Interventions and comparators

A broad range of intervention types and study designs will be considered eligible for inclusion. Intervention types will include but will not be limited to medical interventions such as surgery and/or medication; therapeutic interventions such as physiotherapy or psychological support; work-based interventions such as job redesign or adaptation of working hours; multicomponent interventions comprising multiple elements; and other interventions.

Study designs will include randomised controlled trials, randomised trials with two or more interventions and no control group, non-randomised controlled trials, and prospective and retrospective comparative cohort studies. Studies without a comparison group will be excluded. Due to the range of study designs considered, there will also be several different comparators, including intervention compared to control (e.g. usual care, waitlist control), more intensive intervention compared to less intensive intervention, and/or two different interventions.

#### Outcomes

Studies must measure and report on at least one work-related outcome to be included in this review. Work-related outcomes could include but are not limited to the following: time to return to work, duration of sick leave, work performance, presenteeism, and work ability.

#### Language

Studies will only be included in the review if they are in English.

### Exclusion criteria

Studies will be excluded if the paper is a protocol only with no intervention effects reported or if the paper is in abstract format only. The research team will make attempts to find full texts of abstracts and the results of protocol papers before exclusion, including contacting study authors (twice via email). Multiple publications from the one trial will only be counted as one study (e.g. methods papers, secondary analyses, dissertations, re-published versions of the same paper). Publications from the same study will be checked for additional information (e.g. secondary outcomes, follow-up assessments) that were not reported in the main report of the study.

### Information sources and search strategy

The review will include published, peer-reviewed articles, and unpublished, ‘grey’ literature in the form of theses and dissertations that meet inclusion criteria. The following databases will be searched: PubMed, Embase, Web of Science, Cumulative Index to Nursing and Allied Health Literature (CINAHL), PsycINFO, Centre for Controlled Trials (CENTRAL), and ProQuest Dissertations & Theses Global using terms that relate to (1) road traffic crash (e.g. ‘car’ and ‘accident’), (2) musculoskeletal injury (e.g. ‘neck’ and ‘trauma’), (3) work (e.g. sick leave), and (4) study design (e.g. randomised). An example search is provided in Additional file 2. Searches will be modified slightly to accommodate each database. Searches will be limited to studies in English where possible. Studies only in children will be removed manually during the screening process. No date limits will be used. References of included papers will also be checked for additional papers, as will the authors’ own personal reference libraries.

### Data collection and extraction

Results from each database search will be exported to separate Endnote files (version ≥ X7; Clarivate Analytics, Philadelphia, PA, USA) which will then be combined and duplicates removed. The primary author (CLB) will conduct the searches and remove duplicates. Two authors (including the primary author) will independently review the titles and abstracts against inclusion and exclusion criteria in Endnote; any discrepancies will be discussed between the two authors and where there is no resolution the input of the senior author will be used to finalise discrepancies. Papers that meet inclusion criteria or require further information will be downloaded as full text.

Study information for papers that meet the inclusion criteria will be extracted by the primary author into a table. Study information will include study design, location of the study, local compensation scheme, participant details (including age, gender, job type, compensation status, injury details, and number of participants working pre-injury), intervention components, comparison groups, work-related outcomes, health-related and functional outcomes, predictors of work-related outcomes, and details of the data analysis (e.g. adjustment for covariates, missing data). A second researcher will also extract this data, and the extracted data will be compared with the data extracted by the primary author. Discrepancies will be resolved through discussion or through input of the senior author. Results will be taken from intention to treat analyses when reported. Efforts will be made to obtain additional information of the compensation scheme in place at the location and time the study was delivered if this is not reported in the paper.

#### Primary outcomes

Work-related outcomes are reported below. The following definitions will be used in this review:

- Time until return to work. This refers to the time taken to return to work (in days or weeks) after a period of sickness absence from work. This outcome may be reported as (1) time until first day back at work (i.e. at least one day back at work, [40]), (2) time until a sustained period back at work (e.g. at least 4 weeks back at work [31]), or (3) percentage of participants working at each follow-up point. If specified, this outcome will also be reported as time to full return to work, time to partial return to work (e.g. modified duties), time to return to any work, and time to return to pre-injury work.
- Sick leave. Sick leave can include all sickness absences reported during the study period. This outcome may be reported as (1) cumulative sick leave duration, (2) frequency of sick leave absences, or (3) percentage of participants on sick leave at each follow-up point. This outcome may also be referred to as absenteeism.
- Work or job performance. This outcome refers to a worker’s own job performance and/or performance compared to others. It may be self-reported or supervisor-rated and may be reported as a total score (e.g. out of 10).
- Presenteeism. This outcome refers to when an employee attends work despite poor health [41]. The World Health Organization’s Health and Work Performance Questionnaire is an example of a self-reported measure of presenteeism [42]. Presenteeism may be reported as a total score (e.g. out of 100).
- Work ability or work capacity. This outcome refers to a worker’s ability to do work in relation to work demands, health, and mental resources [43]. This outcome may be self-reported (e.g. the Work Ability Index [43]) or objectively measured (e.g. a Functional Capacity Evaluation [44]). This outcome may be reported as (1) a total score or (2) a category of work ability (e.g. poor, moderate, good).

Time to return to work and sick leave will be considered as separate outcomes because sick leave can take into account reoccurrences of sick leave after initial return to work. Only outcomes reported at the end of the intervention (i.e. the first assessment after all intervention contact has ceased) and any subsequent outcome measurement after this (to assess maintenance of intervention effects) will be reported. If intervention length is unclear, the last assessment point will be reported. Studies will be considered as having a positive work-related outcome if there is a statistically significant (*p* < 0.05) difference between intervention groups at follow-up for at least one of the work-related outcomes. Effect sizes will be calculated where possible, and the effects discussed in terms of their clinical significance.

#### Secondary outcomes

To enhance understanding of the effect of the interventions on work-related outcomes, a select range of secondary outcomes will be reported. These include:

- Pain
- Pain-related disability
- Quality of life
- Physical functioning
- Psychological functioning
- Cognitive functioning
- Social support
- Fatigue
- Return to usual activities
- Return to driving
- Self-reported recovery

Pain outcomes may be reported as (1) a scale on the severity of pain (e.g. a visual analogue scale or numeric rating scale [45]) or (2) the presence of any pain symptoms (yes/no). For quality of life (e.g. the Short-Form 36 [46]), only the total score or physical and mental subscales (if applicable) will be reported in the review.

### Risk of bias

Risk of bias will be measured using the Cochrane risk-of-bias tool for randomised trials [47] and the Risk Of Bias in Non-randomised Studies - of Interventions (ROBINS-I) assessment tool [48] by two authors (including the primary author) independently. Randomised trials will be assessed as ‘high risk’, ‘low risk’, or ‘some concerns’ across five domains. The five domains consist of (1) bias from the randomisation process, (2) bias due to deviations from intended interventions, (3) bias due to missing outcome data, (4) bias in measurement of the outcome, and (5) bias in the selection of the reported result [47]. An additional domain, ‘bias arising from identification or recruitment of individual participants within clusters’, will be included for cluster designs. Non-randomised studies will be assessed as ‘low risk’, ‘moderate risk’, ‘serious risk’, or ‘critical risk’ across seven domains. The domains include (1) bias due to confounding, (2) bias due to participant selection, (3) bias due to intervention classification, (4) bias due to deviations from intended interventions, (5) bias due to missing data, (6) bias in measurement of outcomes, and (7) bias in the selection of the reported result [48]. Discrepancies between the two authors will be discussed, and if the discrepancies cannot be resolved, a third author will be consulted.

All studies will be included in this review regardless of bias, and the bias scores will be used to guide the narrative results and discussion of the findings. The overall strength of the evidence will also be evaluated using the GRADE approach by two authors independently and will consider the following domains: risk of bias findings, consistency of results, precision of results, directness (e.g. the extent that participants, interventions, and outcome measures in the study are relevant to those of interest), and publication bias [49]. The overall strength of the evidence will be rated as high, moderate, low or very low.

### Data synthesis

A narrative synthesis will first be conducted to describe study details (locations, dates of publication, sample sizes, and study designs), participant characteristics (age, gender, job type, compensation status, injury severity, and injury type), intervention characteristics (duration and frequency of contacts, intervention details, interventionist professions, main target of the intervention, and intervention setting), control conditions, compensation schemes, and outcomes of the included studies. If reported by any studies, results from predictor analyses of work-related outcomes will also be summarised. Continuous work-related outcomes (e.g. time to return to work) will be reported as the mean difference (measure of variance) between intervention groups (as well as a standardised measure of effect) and categorical outcomes (e.g. return to work rate at follow-up) will be reported as a risk ratio between intervention groups.

The narrative synthesis will be used to inform the appropriateness of conducting a meta-analysis. A meta-analysis will first be performed using standardised effects, provided that standardised effects of work outcomes can be calculated for at least three studies. Subgroup analyses will then be performed for interventions compared to usual care/control and for interventions compared to interventions. A meta-analysis will also be conducted for each separate work outcome and for each secondary outcome if there are at least three studies reporting on that outcome. For the meta-analyses, RevMan (version ≥ 5.3, [50]) will be used to synthesise the results on the outcomes and produce the forest plots. Random-effects models will be performed as we anticipate high variation across studies. Study heterogeneity will be reported using tau-squared and *I*-squared statistics [51, 52]. Publication bias will be evaluated using funnel plots and, where there are at least ten studies in a meta-analysis, tested using Egger’s test [53] for continuous outcomes and the arcsine-Thompson test for categorical outcomes [51] as recommended by the Cochrane Handbook for Systematic Reviews of Interventions [54] in R (version ≥ 3.6.0; R Core Team). Sensitivity tests using the ‘leave-one-out’ approach will be conducted to examine if the results are dependent on any one study.

To explore objective two of this review, the association of participant characteristics (age, proportion of women, proportion of participants compensated, job type, injury severity, and injury type), intervention characteristics (duration of intervention, duration of follow-up, number of intervention contacts, interventionist profession, intervention setting, intervention type and target [e.g. work-based], and intensity of intervention [e.g. supervised vs. unsupervised]), workplace characteristics (e.g. presence of manager support), and external factors (compensation scheme and study location) with work outcomes will be explored using meta-regression. Meta-regression will only be conducted if there are at least ten studies in the meta-analysis (as recommended by the Cochrane Handbook for Systematic Reviews of Interventions [55]). If any participant characteristic is identified in the meta-regression as associated with work outcomes, we will clarify that the finding is at the study-level rather than the individual-level (e.g. better work-related outcomes are associated with *studies* with participants who have milder injuries) to avoid issues with ecological fallacy. Should there be insufficient data to conduct meta-regression analyses, characteristics will be contrasted between studies with a positive work-related outcome, and those with no positive work-related outcomes in the narrative synthesis.

### Plan for documenting amendments

If there are any amendments to this protocol, these will be highlighted in the published final review and updated in PROSPERO.

## Discussion

This systematic review will explore whether interventions targeting individuals with musculoskeletal injuries sustained in a road traffic crash can improve work-related outcomes and what factors are associated with improved outcomes. This review will provide a concise resource of current evidence suitable for use by policy makers, stakeholders, and researchers and may help inform the design and implementation of interventions in this group. This review may also identify factors that are associated with improved work outcomes. If these factors are participant characteristics, they can be taken into account when designing a screening tool to identify individuals needing more intensive intervention. If the factors are related to the intervention or external characteristics, the results may help to further develop and improve interventions and inform policy. The findings of this review may also identify if there are any research gaps and opportunities for future research. Limitations of this review include the inclusion of English language papers only and including all studies regardless of methodological bias and randomisation status. The implications of the first limitation are that there may be countries and compensation schemes that are less represented in the review, and the findings from this review may be applicable to certain countries only. Including studies regardless of study quality enables exploration of a range of study designs in this population; however, effects should be analysed with caution. Limitations will be discussed further in the full review.

## Supplementary information

**Additional file 1.** PRISMA-P 2015 Checklist.

**Additional file 2.** Planned PubMed search terms. Example search terms to be used in PubMed database.

## Data Availability

Not applicable

## Abbreviations

CENTRAL: Centre for Controlled Trials
CINAHL: Cumulative Index to Nursing and Allied Health Literature
GRADE: Grading of Recommendations Assessment, Development and Evaluation
PRISMA-P: Preferred Reporting Items for Systematic review and Meta-Analysis Protocols
